# Comparison of DNA Extraction Kits for Detection of *Burkholderia pseudomallei* in Spiked Human Whole Blood Using Real-Time PCR

**DOI:** 10.1371/journal.pone.0058032

**Published:** 2013-02-27

**Authors:** Nicole L. Podnecky, Mindy G. Elrod, Bruce R. Newton, Leslie A. Dauphin, Jianrong Shi, Sutthinan Chawalchitiporn, Henry C. Baggett, Alex R. Hoffmaster, Jay E. Gee

**Affiliations:** 1 Bacterial Special Pathogens Branch, Division of High-Consequence Pathogens and Pathology, National Center for Emerging and Zoonotic Infectious Diseases, Centers for Disease Control and Prevention, Atlanta, Georgia, United States of America; 2 Bioterrorism Rapid Response and Advanced Technology Laboratory, Division of Preparedness and Emerging Infections, National Center for Emerging and Zoonotic Infectious Diseases, Centers for Disease Control and Prevention, Atlanta, Georgia, United States of America; 3 Division of Global Migration and Quarantine, National Center for Emerging and Zoonotic Infectious Diseases, Centers for Disease Control and Prevention, Atlanta, Georgia, United States of America; 4 Nakhon Phanom Provincial Health Office, Nakhon Phanom, Thailand; 5 International Emerging Infections Program, Global Disease Detection Regional Center, Thailand Ministry of Public Health-US Centers for Disease Control and Prevention Collaboration, Nonthaburi, Thailand; The University of Texas-Houston Medical School, United States of America

## Abstract

*Burkholderia pseudomallei*, the etiologic agent of melioidosis, is endemic in northern Australia and Southeast Asia and can cause severe septicemia that may lead to death in 20% to 50% of cases. Rapid detection of *B. pseudomallei* infection is crucial for timely treatment of septic patients. This study evaluated seven commercially available DNA extraction kits to determine the relative recovery of *B. pseudomallei* DNA from spiked EDTA-containing human whole blood. The evaluation included three manual kits: the QIAamp DNA Mini kit, the QIAamp DNA Blood Mini kit, and the High Pure PCR Template Preparation kit; and four automated systems: the MagNAPure LC using the DNA Isolation Kit I, the MagNAPure Compact using the Nucleic Acid Isolation Kit I, and the QIAcube using the QIAamp DNA Mini kit and the QIAamp DNA Blood Mini kit. Detection of *B. pseudomallei* DNA extracted by each kit was performed using the *B. pseudomallei* specific type III secretion real-time PCR (TTS1) assay. Crossing threshold (C*_T_*) values were used to compare the limit of detection and reproducibility of each kit. This study also compared the DNA concentrations and DNA purity yielded for each kit. The following kits consistently yielded DNA that produced a detectable signal from blood spiked with 5.5×10^4^ colony forming units per mL: the High Pure PCR Template Preparation, QIAamp DNA Mini, MagNA Pure Compact, and the QIAcube running the QIAamp DNA Mini and QIAamp DNA Blood Mini kits. The High Pure PCR Template Preparation kit yielded the lowest limit of detection with spiked blood, but when this kit was used with blood from patients with confirmed cases of melioidosis, the bacteria was not reliably detected indicating blood may not be an optimal specimen.

## Introduction

*Burkholderia pseudomallei*, a Gram-negative bacterium, is the etiologic agent of melioidosis; a disease with varying severity that can affect both human and animal populations. *B. pseudomallei* is recognized as a Select Agent by the Centers for Disease Control and Prevention based on the criteria of infectivity, severity, and environmental prevalence [1]. Melioidosis is endemic in Southeast Asia and northern Australia but may occur in other tropical regions of the world [2]. In its acute form, melioidosis can manifest as a severe septicemia, which, without prompt diagnosis is often fatal [3], [4]. *B. pseudomallei* is resistant to many of the broad spectrum antibiotics often used for treatment of sepsis [4]. Rapid diagnosis of melioidosis allows for prompt treatment with appropriate antibiotics and thus reduces mortality [4].

Culture of *B. pseudomallei* from any clinical specimen is the current gold standard for the diagnosis of melioidosis. The biochemical analysis may require up to seven days before confirmation [5], [6]. Serologic assays such as enzyme-linked immunosorbent assays (ELISA) or indirect hemagglutination assay (IHA) have been shown to be unreliable when used in regions where melioidosis is endemic due to elevated antibody levels in healthy populations [3], [4], [7], [8]. Many polymerase chain reaction (PCR) assays have been developed for the detection of *B. pseudomallei* from a variety of sources such as clinical specimens, environmental samples and pure culture by targeting a variety of genes [9], [10], [11], [12], [13], [14], [15]. The ability to detect *B. pseudomallei* in whole blood with PCR is dependent on the bacterial load, the quantity and quality of the bacterial DNA extracted and the elimination of PCR inhibitors.

There are many commercial DNA extraction kits that are designed to extract quality DNA and eliminate PCR inhibitors from blood samples. Many of these kits state their use is not intended for clinical or diagnostic applications. However with proper verification, validation and quality control, they can be valuable tools for the detection of pathogens with assays such as conventional and real-time PCR, DNA sequencing, and DNA hybridization assays. Commercial DNA extraction kits are generally available worldwide, complete with standardized methods and reagents, and are simple to use [16].

No DNA extraction method has been shown to be optimal for all bacteria [17]. There have been studies to determine the best method for specific species of bacteria such as *Brucella melitensis*, *Mycobacterium spp.*, *Leptospira spp.*, and most recently *Bacillus anthracis* and *Yersinia pestis* [18], [19], [20], [21], [22]. A study by Merk *et al*. in 2006 used a *B. pseudomallei* surrogate organism, *Burkholderia cepacia*, to compare DNA isolation methods for artificially infected ethylenediaminetetraacetic acid (EDTA)-containing equine blood and lung tissue [23]. This study found that the High Pure PCR Template Preparation kit performed the best. However, while our study is similar, current Clinical and Laboratory Standards Institute (CLSI) guidelines recommend evaluating performance standards for each pathogen instead of using surrogate organisms. Ideally, as part of the assessment process for determining sensitivity and specificity, CLSI recommends using clinical specimens from known cases of a given disease as part of the positive control panel and using material from close relatives of a given pathogen as part of the negative control panel [16].

The purpose of this study was to compare seven commercially available DNA extraction kits to determine which may be best for extracting *B. pseudomallei* genomic DNA from spiked whole blood containing EDTA for detection by real-time PCR. The kits were selected based on their availability in the United States of America, Thailand, and Australia; varying throughput capacities; purification technology and previous use by other laboratories for detecting *B. pseudomallei* DNA. Kits were also selected based on cost due to budget constraints that some laboratories may have.

## Materials and Methods

### Kit Selection

The DNA extraction formats tested include glass or silica filter columns and magnetic bead automated systems which appear to be the most common technologies used in commercial kits. These kits also are considered less hazardous than older methods such as the use of phenol/chloroform extractions which may expose staff to carcinogens. Kits were also chosen based on both availability in areas where melioidosis is endemic or where it poses a potential biothreat concern. In addition some kits were not assessed based on higher costs compared to those that were selected for assessment. The following manual kits were evaluated: QIAamp DNA Mini Kit (QIAamp Mini) (QIAGEN, Valencia, CA), QIAamp DNA Blood Mini Kit (QIAamp Blood) (QIAGEN, Valencia, CA), and the High Pure PCR Template Preparation Kit (High Pure) (Roche Diagnostics, Indianapolis, IN). These kits were compared with four automated systems which provide greater throughput than manual kits: the MagNA Pure LC (Roche Diagnostics, Indianapolis, IN) with the DNA Isolation Kit I (MagNA LC) which can process 32 specimens per run, the MagNA Pure Compact (Roche Diagnostics, Indianapolis, IN) with the Nucleic Acid Isolation Kit I (MagNA Compact) which can process eight specimens per run and the QIAcube (QIAGEN, Valencia, CA) using both the QIAamp DNA Mini kit (QIAcube Mini) and QIAamp DNA Blood Mini kit (QIAcube Blood) which has a capacity of 12 specimens per run.

### Bacterial Strains

The *B. pseudomallei* type strain, ATCC 23343, was used in this study. All work done with *B. pseudomallei* live cultures was performed in a biosafety level 3 (BSL-3) facility following BSL-3 safe practices and procedures.

### Blood Spiking

Human whole blood for this experiment was obtained in Vacutainer K2 EDTA tubes (BD Diagnostics, Sparks, MD) from an anonymous donor through the Specimen Management Branch of the CDC. *B. pseudomallei* bacteria were suspended in phosphate- buffered saline (PBS) to between a 1 and 2 McFarland standard, estimated to be 4.5×10^8^ colony forming units per milliliter (CFU/mL). The whole blood with EDTA was spiked to 4.5×10^7^ CFU/mL from this PBS solution for a 1∶10 dilution, then serially diluted in blood by 10-fold to a theoretical concentration of less than 1 CFU/mL. An aliquot of 100 μl from each dilution was plated on trypticase soy agar containing 5% sheep blood (TSA II; BD Diagnostics, Sparks, MD) in triplicate and incubated at 37°C for 48 hours for a more accurate enumeration than the estimate yielded by using the McFarland standard. Due to the number of kits tested and time constraints, all dilutions of the spiked blood samples were stored frozen for each of the kits at −70°C. An aliquot of a given dilution was thawed to room temperature just prior to processing by a given kit.

### DNA Extractions

The serial diluted spiked blood samples, as well as the negative blood and negative water controls were extracted in triplicate using seven different DNA extraction kits as follows. The two manual QIAGEN kits tested in this study, the QIAamp Mini and the QIAamp Blood, were used following the manufacturer's instructions for the blood and body fluid spin protocol. A 200 μl blood sample was extracted, the optional spin at 20,000×*g* for 1 min prior to incubation and elution with Buffer AE was performed, and 95% ethanol was used instead of the manufacturer's recommended 96%–100% ethanol. A separate short study indicated no significant difference in using 95% ethanol compared to 99.5% ethanol (data not shown). Additionally, these two QIAGEN kits were used with the QIAcube automated system following the manufacturer's instructions for sample setup of the QIAcube Mini and QIAcube Blood kits. The High Pure kit was used following the manufacturer's instructions for 200 μl of mammalian blood. The MagNA Compact utilized the Nucleic Acid Isolation Kit I (Roche Applied Science, Indianapolis, IN), which contains all necessary reagents and disposables. To optimize DNA recovery and enhance cell deactivation, an optional external lysis protocol was utilized prior to the automated MagNA Compact extraction using the DNA Blood External Lysis Purification protocol. This included combining 200 µl of the blood sample with 300 µl of the MagNA Pure LC DNA Isolation Kit I – Lysis/Binding Buffer, mixing, and incubating at room temperature for 30 min. The MagNA LC utilized the MagNA Pure LC DNA Isolation Kit I and the same external lysis was completed, as described above, and DNA extracted using the DNA Blood External Lysis Purification protocol, as described above. The positive control, for the real-time PCR detection, was a whole-cell lysate of *B. pseudomallei* (ATCC 23343) produced as described previously by Hoffmaster *et al*. [24], which provided crossing threshold (C*_T_*) values ranging between 23 and 28 cycles. As a precaution all DNA extracts were filtered using 0.22-µm centrifugal filter units (Millipore, Corporation, Billerica, MA) and then an aliquot was plated to assess removal of viable cells. All extracted DNA samples were stored at −20°C in their provided elution buffers, until analyzed by real-time PCR. Both water and unspiked blood were processed alongside spiked blood and served as extraction controls to determine if cross contamination occurred.

### Real-time PCR Detection

Detection of *B. pseudomallei* DNA in the extracted blood samples was performed using the *B. pseudomallei* type III secretion system (TTS1) real-time PCR assay with a SmartCycler II instrument (Cepheid, Sunnyvale, CA) using the LightCycler FastStart DNA Master HybProbe (Roche Diagnostics, Indianapolis, IN) PCR master mix as previously described by Novak *et al*. [13]. The following modifications were made to the TTS1 assay: 5 μl of template was used in a final reaction volume of 25 μl and an alteration of the cycling parameters was made to increase the extension time from 15 s per cycle to 60 s per cycle.

### Comparison of DNA Extraction Kits

The seven commercially available DNA extraction kits were compared to determine their lower limit of detection. In order to evaluate the recovery efficiency, each DNA extraction set was run by the TTS1 real-time PCR assay in triplicate. This provided a total of nine crossing threshold (C*_T_*) values, as determined by the Smart Cycler Dx® program (Cepheid, Sunnyvale, CA; Software Version 1.7b), for each spiked blood dilution per extraction kit. The C*_T_* values were used to determine the reproducibility of results and the detection limit for each kit. The detection limit for each DNA extraction kit was decided to be the lowest spiked blood concentration at which 100% of the samples yielded positive results. A 1 μl sample from each spiked blood DNA extraction including the blood not spiked with bacteria was analyzed using the NanoDrop ND-1000 spectrophotometer (Thermo Scientific, Wilmington, DE, USA) in triplicate and the results averaged to determine the DNA concentration, the absorbance at 260 nm (*A_260_*), of the samples from each extraction kit. Additionally, the absorbance at 280 nm (*A_280_*) was measured and the *A_260_*/*A_280_* ratio determined to evaluate DNA purity. An average was taken for all the samples extracted from each kit to determine the average *A_260_* value and *A_260_*/*A_280_* ratio. To determine if PCR inhibitors were being recovered along with DNA, DNA extracts of non-spiked blood from the QIAamp Mini, QIAamp Blood, High Pure, MagNA LC and MagNA Compact kits were mixed with positive control DNA in a 10∶1 ratio and tested for changes in C*_T_* values compared to the positive control DNA combined with H_2_O.

### Testing on Clinical Blood Specimens

Eleven blood specimens from blood culture-confirmed cases of melioidosis from Sa Kaeo and Nakhon Phanom Provinces, Thailand were collected along with five blood specimens from patients with *E. coli* septicemia as negative controls. Blood cultures were collected at clinician discretion and were likely collected at the time of admission. The blood specimens tested were collected as part of a pneumonia study and patients were enrolled up to 24 hours after admission. The patients probably received antibiotics during the time between blood culture and collection of the blood specimen tested as part of this evaluation. Specimens were relabeled prior to processing so that the laboratorians were blinded to origins of specimens and were processed using the Hi Pure kit and tested using the TTS1 PCR assay. The blood specimens were collected as part of a pneumonia etiology study approved by a CDC Institutional Review Board and the Ethical Review Committee of the Thailand Ministry of Public Health.

### Statistical Analysis

To determine the reproducibility of the DNA extraction kits, we analyzed experimental data from a balanced replicated design with two factors, the type of extraction kits and the concentration, using a two-way analysis of variance (ANOVA). This allowed us to compare the effects of changing DNA extraction method and the concentration on expected C*_T_* values. Pair-wise comparisons of C*_T_* values for the DNA extraction kits were done using the Tukey multiple comparisons test [25]. All analyses were performed using the statistical software SAS 9.3 (SAS Institute, Cary, NC). All tests of statistical significance were two-sided, and the significance level was set at 5%.

## Results

*B. pseudomallei* bacteria were used to generate serial dilutions of spiked whole blood samples. By the plate count method the concentrations of bacteria in the spiked samples ranged from 5.5×10^6^ CFU/mL down to an undetectable level. These serially diluted samples were used for testing and comparison of the performance of the seven DNA extraction kits.

The highest average DNA concentration was 51.55 ng/µl from samples extracted by the QIAcube Blood, while the MagNA LC extractions had the lowest DNA concentrations at an average of 4.90 ng/µl (Table 1). The MagNA Compact and the QIAcube Mini had lower DNA concentrations of 16.37 ng/µl and 17.62 ng/µl, respectively, while the remaining kits had average yields greater than 30 ng/µl. There was no observable correlation in DNA concentration with the addition of bacteria, which is not unexpected due to the relatively large mass of DNA provided by human cells compared to the bacteria added (data not shown). The QIAcube Mini, QIAcube Blood, and the MagNA Compact extracted DNA samples had *A_260_*/*A_280_* ratios that were between 1.7 and 1.9. The other DNA extraction kits all yielded samples with *A_260_*/*A_280_* ratios around 2.0 except for the MagNA LC, which had a ratio of 2.25.

**Table 1:** Average concentration and purity of DNA extraction performed by commercially available DNA extraction kits on *Burkholderia pseudomallei* spiked whole blood containing EDTA.

DNA Extraction Kit	DNA Concentration ng/µl	DNA Purity A*_260_*/*_280_*
QIAcube Blood	51.55	1.83
QIAamp Mini	38.05	2.06
QIAamp Blood	30.49	1.98
High Pure	30.01	2.07
QIAcube Mini	17.62	1.79
MagNA Compact	16.37	1.77
MagNA LC	4.90	2.25

Averages based on all blood specimens.

In this study, C*_T_* values using the TTS1 real-time PCR assay were used to compare the limit of detection and reproducibility of each kit. As shown in Table 2, the PCR limit of detection using DNA extracted by the different kits varied 1000 fold, from 5.5×10^3^ to 5.5×10^6^ CFU/mL. The High Pure kit yielded DNA extractions that resulted in the lowest limits of detection, 5.5×10^3^ CFU/mL at 100% of the time, and additionally was detected at 4.9×10^2^ CFU/mL at a frequency of roughly 11%. The QIAamp Mini, QIAcube Blood, QIAcube Mini, and the MagNA Compact all yielded DNA preparations that were detected at various frequencies at 5.5×10^3^ CFU/mL and 100% of the time from the spiked blood samples at concentrations of 5.5×10^4^ CFU/mL and greater. However, both the QIAamp Blood and the MagNA LC had the higher limits of detection at 5.5×10^5^ CFU/mL, and 5.5×10^6^ CFU/mL, respectively.

**Table 2:** Comparison of seven DNA extraction kits based on the average crossing threshold (C*_T_*) values, standard deviation and number of PCR positives using the TTS1 real-time PCR protocol.

*B. pseudomallei* CFU/mL	5.5×10^6^		5.5×10^5^		5.5×10^4^		5.5×10^3^		4.9×10^2^	
	Avg. C*_T_* (SD)	# PCR+	Avg. C*_T_* (SD)	# PCR+	Avg. C*_T_* (SD)	# PCR+	Avg. C*_T_* (SD)	# PCR+	Avg. C*_T_* (SD)	# PCR+
High Pure	22.8 (0.4)	9/9	26.3 (0.7)	9/9	29.6 (0.6)	9/9	33.5 (0.4)	9/9	37.3 (0.0)	1/9
QIAamp Mini	24.8 (0.9)	9/9	27.3 (1.3)	9/9	30.0 (1.1)	9/9	35.9 (2.8)	7/9	−	0/9
QIAcube Blood	24.9 (0.8)	9/9	29.0 (0.6)	9/9	31.7 (0.4)	9/9	36.4 (1.1)	7/9	−	0/9
QIAcube Mini	25.4 (0.2)	9/9	29.4 (0.1)	9/9	32.9 (0.4)	9/9	38.3 (1.0)	5/9	−	0/9
MagNA Compact	26.1 (0.4)	9/9	28.9 (1.1)	9/9	32.6 (0.8)	9/9	34.8 (0.6)	2/9	−	0/9
QIAamp Blood	24.7 (1.1)	9/9	28.0 (0.7)	9/9	31.3 (1.0)	8/9	−	0/9	−	0/9
MagNA LC	29.1 (2.8)	9/9	33.8 (0.9)	8/9	34.8 (1.8)	4/9	−	0/9	−	0/9

CFU/mL  =  Colony forming units per milliliter; SD  =  Standard deviation.

The differences in the mean C*_T_* values for the seven DNA extraction methods were found to be statistically significant by the ANOVA test (*P*<0.05). The Tukey multiple comparison test indicated that the High Pure kit was the DNA extraction kit that yielded DNA providing C*_T_* values significantly lower than any of 6 other DNA extraction kits (*P*<0.05), after pairwise comparisons.

A test was performed using available DNA extracts from non-spiked blood combined with positive control DNA. If inhibitors were not removed, one would expect to see an increase in C*_T_* values compared to the positive control DNA diluted with water. No significant difference in C*_T_* values was observed (data not shown). Extraction controls remained negative throughout the study (data not shown).

Based on the performance of the High Pure kit with spiked blood, we chose to evaluate it using 11 blood specimens from patients in Thailand with confirmed cases of melioidosis. None yielded amplification on all three of the triplicates using the TTS1 assay. However, one specimen indicated amplification on two of the three triplicates. Three specimens had one of the triplicates indicate amplification. C*_T_* values were at 40 or above. None of the negative controls from the *E. coli* infections indicated amplification (data not shown).

## Discussion

*B. pseudomallei* infection can cause septicemia which is fatal in roughly 50% of adult patients in Thailand, while the mortality rate is about 20% in Australia [4], [26]. Although broad spectrum antimicrobial treatment is often initiated prior to bacterial identification, *B. pseudomallei* infection, which can mimic other infections, is resistant to treatment by many commonly used antimicrobial agents [4]. Rapid diagnosis of infection could expedite administration of appropriate antimicrobial therapy and thereby potentially improve survival rates. Real-time PCR assays have the capability to identify *B. pseudomallei* infection within hours. Real-time PCR and other DNA-based methods for diagnosis are dependent on the quality and timeliness of the DNA extraction process.

Of the seven commercially available DNA extraction kits tested in this study the High Pure kit yielded DNA extractions that resulted in the lowest limits of detection, 5.5×10^3^ CFU/mL at 100% of the time, and additionally was detected at 4.9×10^2^ CFU/mL at a frequency of roughly 11%. As a result of pairwise comparisons, the High Pure kit was the DNA extraction kit yielding the lowest C*_T_* values statistically (*P*<0.05).

If the Tukey multiple comparison test was run at three high concentrations (5.5×10^4^, 5.5×10^5^ and 5.5×10^6^ CFU/mL), the High Pure kit still had the lowest C*_T_* values statistically.

(*P*<0.05), as compared to six other DNA extraction kits including QIAamp Blood. This indicates the consistently lowest values for each of the diluted samples that were detected. The QIAamp Mini, QIAamp Blood, MagNA Compact, QIAcube Mini, and QIAcube Blood Mini kits yielded DNA extractions that resulted in a limit of detection of 5.5×10^4^ CFU/mL, and additionally were detected at 5.5×10^3^ CFU/mL at varying frequencies.

The DNA concentrations determined by the Nanodrop ND-1000 spectrophotometer indicate the total DNA extracted from the whole blood specimen, which includes the bacterial DNA. The majority of the DNA in the extracted samples is expected to be from the whole blood itself due to the relatively large mass of blood cells compared to the bacterial cells added. Thus it may not be possible to predict detection of *B. pseudomallei* DNA based on the DNA concentration, as the MagNA Compact had only 16.37 ng/µl but had the same limit of detection as the manual QIAamp Mini, which yielded 38.05 ng/µl. The DNA purity, *A_260_*/*A_280_* ratio, was around 2.00 for the kits that performed best in this study: the High Pure, QIAamp Mini, QIAcube Mini, QIAcube Blood, and MagNA Compact. While there is no set standard for what the optimal *A_260_*/*A_280_* ratio is, it has generally been stated that an *A_260_*/*A_280_* ratio between 1.8 and 2.0 is considered to be free of significant contamination [27].

The study done by Merk *et al*. in 2006 found that the High Pure kit had the lowest limit of detection for EDTA equine blood that was spiked with *B. cepacia* [23]. No other kits or DNA extraction methods were assessed by their study and the current study. Merk *et al*. determined the detection limit for the High Pure kit to be between 2.6×10^3^ and 6.4×10^4^ CFU/mL, which is consistent with our results indicating the detection limit to be 5.5×10^3^ CFU/mL. There were a few differences in between these two studies; Merk *et al*. centrifuged the whole blood samples and extracted DNA from the pelleted samples and utilized conventional PCR [23].

The ability to extract sufficient *B. pseudomallei* DNA from whole blood samples for detection by PCR would be an important capacity for the rapid diagnosis and proper treatment of septic melioidosis. This capacity would be most beneficial where *B. pseudomallei* infection is endemic. In these regions the use of commercial DNA extraction kits have many advantages including the availability of the kits, ease of use, relative low cost and time required, and standardization of the protocols and reagents. Although the High Pure kit yielded the lowest limit of detection for blood samples spiked with *B. pseudomallei*, other kits may offer other advantages. For example, the other manual kits may have lower associated costs for disposables and supplies. The High Pure kit requires heating of the elution buffer which is not required by some other manual kits such as the Qiagen kits. The other manual Qiagen kits as well as the QIAcube Blood yielded higher total DNA concentrations which may be advantageous if other PCR or genetic testing will be performed for a given specimen. Manual kits are labor intensive and are more prone to technician error, while the automated kits take the same, if not more, time to perform a single extraction, but require less time for set-up by laboratory personnel and can increase throughput. However, testing of only one or a few samples using automated systems may be costly and wasteful. Manual kits are more flexible in the number of samples that can be extracted, and samples can be tested individually upon arrival in a time-sensitive scenario. The initial cost of setting up automated systems is also more due to the need to purchase the robot system to run the kits. However, automated systems would result in relatively lower labor costs if large numbers of specimens were to be processed.

A recent study in Thailand was done to better understand the concentration of *B. pseudomallei* in different body fluids from infected individuals. This study found that the median concentration of *B. pseudomallei* bacteria in blood from culture confirmed cases is 1.1 CFU/mL and the greatest count was over 100 CFU/mL [28]. Other studies have reported ranges from 1 to 1000 CFU/mL in septicemic patients' blood [6], [29], [30]. Since the limit of detection determined for the TTS1 real-time PCR with spiked whole blood extracted using the High Pure kit was 5.5×10^3^ CFU/mL, it may still be difficult to detect septic melioidosis by DNA extraction and TTS1 real-time PCR. However, a small study on clinical specimens with the TTS1 detection system was conducted by Meumann *et al*. [31], who found that while the buffy coat from blood samples extracted with the QIAamp DNA Mini Kit had decreased sensitivity compared to other sample types tested, the method was more successful than previous PCR methods. They were able to detect *B. pseudomallei* in 56% of blood culture positive samples and 17% of blood culture negative samples from patients with confirmed melioidosis [31]. A more recent study by Richardson *et al*. on clinical specimens from melioidosis patients found that Qiagen QIAamp kits worked best using plasma. Interestingly, they found that sputum and urine were the best specimens. Their study did not include the Hi Pure kit [32]. It is not possible to test all commercial kits available based on costs, personnel constraints and limits of specimen availability. Laboratories that perform multiple tests on a given specimen may find that the higher DNA yields of some kits such as the QIAcube Blood may be advantageous compared to the Hi Pure kit. Also, the higher throughput of the automated systems may be advantageous compared to the manual labor required for the Hi Pure kit.

Our attempt to detect *B. pseudomallei* in blood specimens from confirmed cases of melioidosis using the Hi Pure kit did not yield amplification on all three of the triplicates for any of the culture confirmed specimens tested even though this kit had the lowest limit of detection on spiked blood. The failure could be due to low levels of bacteria very near the threshold of the limit of detection which may have been exacerbated by the start of antimicrobial therapy prior to the blood draw. The timing of antimicrobial therapy for these patients is not available.

As other studies have shown, PCR assays are highly specific methods for detection but do need improvements in sensitivity [33]. One strategy to improve the limit of detection of this assay would be to centrifuge the whole blood samples and perform the DNA extraction on the blood fraction that contains the concentrated bacteria, as was done in the study by Merk *et al*. [23], or performing the DNA extractions from a larger sample volume. Testing of other specimen types such as sputum samples and wound cultures have also shown significantly improved rates of detection for *B. pseudomallei* as compared to blood samples [31]. Further studies, looking at a variety of clinical specimens from patients with confirmed melioidosis is needed to see if these rapid methods can significantly reduce the time for diagnosis. This study illustrates the differences in performance of DNA extraction methods as well as other variables to consider during molecular assay development for the detection of specific pathogens. It also further supports previous studies indicating the difficulty in detecting *B. pseudomallei* in blood specimens.
